# Bodily Endurance

**Published:** 1857-04

**Authors:** 


### BODILY ENDURANCE.

An ecclesiastic whose keenness of logic, whose thorough scholarship, whose depth of thought and breadth of view, have made his name familiar to both hemispheres, in a private letter, gives us credit for possessing a sounder theology than half the ministers in the land. May be he had not learned that we have considered it a self-evident proposition that the human heart was the seat of a depravity all-pervading. In that respect we are John Calvin, and if anything different, with a bend backwards. We do not believe that every human heart is equally bad; some are worse than others, incalculably worse, just as of several glasses of pure water, a few drops of ink will color the whole body of water in one glass, making it totally discolored—not an atom of it that is not colored some; a few additional drops will give a more distinct coloring to the next glass, so that of each glass it may be said, as to the water within it, it is totally discolored, yet some are of a deeper black than others; but all are blackened—every particle of each glass is discolored. No atom of any glass is clear, so no one outgoing of the human heart, in its natural state, is clear, is pure, is without a stain. But the extent of that stain, the depth of its blackness, has a strong exhibition in one of the British Reviews, republished in New York, by Leonard Scott & Co. The article is entitled "*Christian Missions a Failure.*" That is to say, all the money expended by missionaries for the purpose of enabling the heathen to read the Bible, has been a bad investment, that the effort made to enlighten the nations for a century or two past, has "*cost more than it comes to*"—the good done has not been commensurate with the money expended.

We can scarcely conceive of a piece of more virulent, ill-natured malignity than that which must have pervaded the heart of the writer at the time of his penning the article. We can all appreciate the feeling which prompts the using of a dagger—deliberate, determined, vengeful, murderous! We would handle such a one in this way: Your composition shows that you are highly educated, that your associations have been of an elevated character, and that you would shrink from making yourself liable to the charge of being wanting in gentlemanly bearing or honorable dealing. But none of this money was yours, not a cent of it. The persons who made that money, appropriated it willingly in the direction of an object which you yourself admit is desirable. Do you think it altogether proper for one gentleman to dictate to another how he shall spend his own money, or when he has spent it, to inform him that it was improperly done, and hint that it would have been a great deal better, if he had appropriated it in a different direction? Intermeddling, an officious interference with the pecuniary expenditures of a neighbor, of a fellow-citizen, dictating to him as to its appropriation—what is it? What would *you* do in the premises?

Here are a number of people who are anxious that certain persons, strangers to them, should be taught how to read the Bible, thinking that it would promote their happiness; and thus thinking, they, with a noble consistency, use their own money largely to purchase the Bibles, and to send persons to teach how to use them; and here is a man in Scotland, a cultivated scholar, raised in the bosom of the Church, engaging without fee or reward, in an effort to throw ridicule on the attempts of those benevolent men; and in order to make his shafts more efficient, falsifies history, falsifies fact. Verily, we can scarcely imagine, under all the circumstances, a greater depth of innate malignity against the Christian religion. There is one man totally depraved, and the depth of the blackness is unmistakable. The great burden of Bible teaching is love to all human kind, industry in all human callings, temperance in all human enjoyments, and unflinching justice in all human transactions; a book which encourages no wrong-doing—which winks at no vice, tolerates no crime; and here is a man who seeks to thwart the efforts of nobler hearts to make this book available to the millions of our earth, who else will die without its sight—opposing these efforts on the ground that they cost too much money, not a dollar of which was his. How deeply dark, how unfathomably mean, must that man's heart be! what a disgrace to the noble land which gave him birth! May he live to feel ashamed of all that he has written.

So far from Christian Missions being a failure, one single individual within a single lifetime has been the means of initiating instrumentalities which will do more towards breaking up the slave trade, than have the fleets of the three greatest nations on the globe for the last quarter of a century; a single individual, by shutting himself out of civilized society for eighteen years, consorting with savages, traversing deserts, swimming rivers, torn by wild beasts, famished by want, and tortured by fiercest fevers, has opened a door to the civilization of a whole continent, occupied by millions of human beings, of whose existence the world never dreamed—an inteterior continent, with its fruitful plains, and navigable rivers, and rich forests—the people themselves comparatively harmless, friendly, and docile; and this man is a Christian missionary, a physician—Dr. Livingston, who has "endured more anxious moments, experienced difficulties and perils, and performed grander and more noble deeds, than any Crimean hero," of whom the Earl of Shaftsbury declared "his great researches and operations will be followed by great and mighty benefits to the whole human race," while Col. Sir R. H. Rawlinson, the learned oriental traveller, expressed his belief that Dr. Livingston had laid the train which would raise interior Africa, with its untutored millions, from the depths of savage degradation.

This unpretending missionary has made himself old in forty years; his face is furrowed by hardships and thirty fevers, and literally black by exposure for sixteen years to an African sun; his left arm crushed and made helpless by a ferocious lion. Having passed through all these privations, he made a journey of a thousand miles on foot, and then further on into an unknown country, stopping not until he had added to his discoveries that of a river navigation of two thousand miles. And while he has done so much for humanity, at so much personal toil and suffering, here is a Scotchman in scholastic Edinburgh, who quietly sits down in his own study and writes "*Christian Missions a Failure*"—"cost more money than the benefits attained pay for."

The life of the great missionary presents several features of physiological interest.

1st. The constitution of man adapts itself to all climates.

2d. The hardships which the human body can endure are incredible until seen, and when encountered without the use of spirituous liquors, leave the constitution as firm and as capable of new endurances, as it was at the beginning.

3d. In all great undertakings requiring persistent endurance of toil, and privation, and exposure, those are most likely to succeed who discard alcoholic drinks of every description, and make up their minds to the temperate indulgence of all the appetites.

4th. Systematic temperance in eating and drinking is capable of shielding the human body from the pestilences of all climes, and from the fatal diseases of all latitudes.

5th. That the hardships which great travellers are called to encounter, do, by their large exposure to out-door air and daily bodily activity, consolidate the constitution, and make it more healthy, while the mental powers take their share of in creased vigor and activity.

